# Rapid, collaborative generation and review of COVID-19 pandemic-specific competencies for family medicine residency training

**DOI:** 10.36834/cmej.70254

**Published:** 2020-09-23

**Authors:** Eric Wooltorton, Edward Seale, Denice Lewis, Kendall Noel, Clare Liddy, Gary Viner, Lina Shoppoff, Douglas Archibald

**Affiliations:** 1 Department of Family Medicine, University of Ottawa, Ontario, Canada

## Abstract

**Background:**

In March 2020, the COVID-19 pandemic disrupted competency-based medical education in Family Medicine programs across Canada. Faculty and residents identified a need for clear, relevant, and specific competencies to frame teaching, learning, supervision and feedback during the pandemic.

**Methods:**

A rapid, iterative, educational quality improvement process was launched. Phase 1 involved experienced educators defining gaps in our program’s existing competency-database, reviewing emerging public health and regulatory guidelines, and drafting competencies. Phase 2 involved translation, member-checking, and anonymous feedback and editing of draft competencies by residents and other educational leaders. Phase 3 involved wider dissemination, collaborative editing and feedback from residents and faculty throughout the department.

**Results:**

A total of 44 physicians including residents and faculty from multiple contexts provided detailed feedback, review, and editing of an ultimate list of 33 competencies organized by CanMEDS-FM roles. Broad agreement was obtained that the competencies form reasonable learning outcomes during the COVID-19 pandemic.

**Conclusions:**

These competencies represent learning objectives reflecting the initial educational mindsets of a wide range of teachers and learners experiencing a global pandemic. The project illustrates a novel collaboration across educational portfolios as a rapid educational response to a public health crisis.

## Abstract (fr)

**Contexte:**

En mars 2020, la pandémie de la COVID-19 a perturbé la formation médicale basée sur les compétences des programmes de médecine familiale partout au Canada. Le corps professoral et les résidents ont identifié la nécessité d’avoir des compétences claires, pertinentes et précises pour encadrer l’enseignement, l’apprentissage, la supervision et la rétroaction durant la pandémie.

**Méthodes:**

Un processus rapide et itératif d’amélioration de la qualité de l’éducation a été lancé. Au cours de la Phase 1, des éducateurs d’expérience ont identifié les lacunes en lien avec la base de données actuelle des compétences du programme, et fait une ébauche de compétences à partir d’une revue des lignes directrices émergentes en santé publique et des organismes de régulation. La Phase 2 a consisté en la traduction, la révision par les membres ainsi que la rétroaction anonyme et la révision des compétences provisoires par les résidents et autres leaders en éducation. Durant la Phase 3, on a procédé à la diffusion à plus large échelle, à la révision en collaboration et au recueil des commentaires des résidents et du corps professoral dans tout le département.

**Résultats:**

En tout, quarante-quatre (44) médecins comportant des résidents et des membres du corps professoral de multiples contextes, ont fourni une rétroaction détaillée et procédé à l’examen et à la révision d’une liste finale de 33 compétences classées par rôles CanMEDS. Une très vaste majorité a convenu que les compétences produisent des résultats d’apprentissage raisonnables durant la pandémie de la COVID-19.

**Conclusions:**

Ces compétences représentent des objectifs d’apprentissage qui reflètent la perspective éducative initiale d’une vaste gamme de d’enseignants et d’apprenants aux prises avec une pandémie mondiale. Le projet représente une nouvelle collaboration entre les programmes d’études comme une réponse éducative rapide à une crise de santé publique.

## Introduction

The bilingual University of Ottawa family medicine program trains family medicine specialists who are competent to provide comprehensive, compassionate care in any Canadian community.^1^ The exceptional circumstances of the COVID-19 pandemic disrupted health systems internationally and forced sudden changes to many training programs including our own in mid-March 2020. Clinical experiences were cancelled or altered abruptly, virtual visits and supervision replaced in-person contact, and resident physicians were re-deployed to novel clinical contexts. For example, residents in our program return for a weekly family medicine ‘half day back’, regardless of their current clinical rotation, allowing a reconnection with a generalist family medicine mindset and practice, and continuity of care and education. However, in March 2020, anticipated surges in patient volumes and physician illnesses and quarantine, and an attempt to minimize clinicians moving between clinical contexts (reducing potential virus spread) led to several major disruptions. Half-day backs were cancelled, electives, leave and teaching and examinations (including the spring certification examination by College of Family Physicians of Canada) were postponed or cancelled, and some residents were re-deployed to under-resourced settings which had not previously hosted residents. Clinical care in family medicine blocks changed dramatically with physical distancing and personal protective equipment requirements, minimal in-person visits (with reduced physical examination) and patient care delivered mostly virtually (phone or video conference), and physicians working remotely and often indirect supervision. All physicians faced new personal and professional challenges. In response to these changes, we launched an adapted, rapid consensus process to identify and define specific COVID-19 related competencies to guide teaching, learning, and feedback in the new clinical reality simultaneously affecting all Departments of Family Medicine across Canada.

## Methods

The uOttawa family medicine program is a bilingual (English, French) two-year residency, with a total of 138 residents (PGY1, PGY2) assigned primarily to one of several dozen of community-based practices across Eastern Ontario, or one of seven teaching units (affiliated with five hospitals in the Ottawa region and surrounding rural areas). Each resident is assigned a faculty preceptor, who are overseen by educational leaders who report on resident progress monthly to a variety of Departmental of Family Medicine committees.

We aimed to engage as many residents, faculty members and leaders in the Department as possible using an adapted approach based on the first three steps of the Kern model^2^: problem identification and general needs assessment (step 1), targeted needs assessment (step 2); writing goals and objectives (step 3) (or more specifically learning ‘outcomes’ in this case^3^); CanMEDS is a one of the most widely used educational frameworks for organizing health professions competencies^4^^-^^6^ and was chosen to organize competencies to allow their use in other Canadian family medicine programs, and non-family medicine specialties (Royal College of Physician and Surgeons of Canada). Traditional group consensus methods, such as Delphi and Nominal group methods^7^ were considered inadequate to meet our urgent timeline to allow for the broad inclusivity needed to capture the contextual relevance of our diverse family medicine training environments and to allow for the timely application of the educational product. Instead, a process of writing, internal peer review, and revision, based on the first three steps of the Kern approach was utilized.^2^ The competencies created are really learning outcomes^5^ which are specific, and observable and include cognitive (knowledge), affective (attitudinal), and psychomotor (skill and behaviours) outcomes for residents, patients, the health care system, and society.

Table 1 provides a summary of the timeline used for the three-phase process that was conducted over a three week period. Rapid iterations of consultations with multiple forms of feedback allowed input from expanding groups of reviewers (faculty, residents). Anonymous feedback through multiple choice, and open-ended questions was tracked in a six-question survey tool (Google Form), with four questions covering basic respondent demographics (to ensure responses from a range of respondents), and open-ended questions collecting suggested changes. *A priori*, we decided we would progress to the next phase if >90% of respondents agreed or strongly agreed with the statement on a 5 point Likert scale (anchors: “strongly agree”, “agree”, “neutral”, “disagree”, “strongly disagree”). In the phase 3, 95.7% of respondents “agreed” or “strongly agreed” with the statement “As a whole the competencies form reasonable learning outcomes to guide our teaching, learning and feedback for residents during the COVID-19 pandemic.”

**Table 1:** Three-phase process to define novel competencies related to Covid-19 Pandemic

Phase and Timing	Participants	Activity Description
Phase 1: “Straw Dog Creation” 1 week	Faculty Development Director Postgraduate (PG) Education Directors (current and former) Curriculum Director Evaluation Directors (current, former) Chair of Family Medicine Director of Undergraduate Education Director of Research	Define emerging challenges facing residents and faculty (needs, gaps) working in multiple contexts via discussions over email, video-conferences Review of emerging guidelines, educational policies and health standards+ Identify gaps in existing curricula, educational tools and competency database Adapt competencies or create them *de novo* using principles consistent with competency-based curriculum development (e.g. relevant, observable outcomes) [5-8] Circulate draft competencies by email in a Word document Incorporate edits and feedback (non-anonymous) through email, and verbally over conference calls
Phase 2: “Input & Clarity Check” 1 week	PG Directors (from each of the 7 urban and rural teaching units and community-faculty) Chief resident physicians Invited faculty and residents from diverse educational contexts	Translate draft competencies Solicit non-anonymous feedback (verbally over conference calls and in writing email, collaborative writing document - Google Doc) and anonymous online surveys (6 question feedback tool - Google Form) Adapt document to incorporate suggested changes or concerns
Phase 3: “Broad Input & Dissemination” 1 week	All resident physicians Faculty clinical leads CFPC PG directors nationally CFPC Faculty Development Education Committee	Create PDF version of competencies to improve ease of reading and review Solicit non-anonymous feedback (email, collaborative writing document - Google Doc – and verbally over conference calls) and new anonymous online surveys (6 question feedback tool - Google Form) Adapt document to incorporate suggested changes or concerns

+ College of Physicians and Surgeons of Ontario, Canadian Medical Protective Association, Canadian Medical Association, Ontario Medical Association, Public Health Agency of Canada, Professional Association of Residents of Ontario, and policies from uOttawa Faculty of Medicine.

The project was granted a signed Research Ethics Board review exemption, and is a registered quality improvement project with the Ottawa Health Science Network Research Ethics Board (OHSN-REB).

## Results

The process and results are described in Table 2. A total of 33 COVID-19 pandemic-specific competencies were created by the end of the departmental process (Figure 1; see supplemental data). In phase three, anonymous feedback (*n* = 24) was provided by residents (*n* = 11, 45.8%) and faculty (*n* = 11, 45.8%) with a range of career experience (29.2% <5 years, 16.7% 5-20 years, 41.7% >20 years) in rural and urban teaching sites. In the phase 3, 95.7% of respondents “agreed” or “strongly agreed” with the statement “As a whole the competencies form reasonable learning outcomes to guide our teaching, learning and feedback for residents during the COVID-19 pandemic.”

**Table 2:** Results of the process

Phase, # respondent reviewers	Results	Comments
Phase 1: ‘Straw Dog Creation’ *n* = 8	Existing competencies in our Department’s curriculum database were often not-specific enough to address the Covid-19 particular challenges A rough list of existing competencies was adapted or created to address the new realities Gaps related to all CanMEDS-FM roles especially the Professional, Health Advocate and FM-expert roles	*Clarification of project’s overall goal, to*: Define competencies for resident physicians related to the COVID-19 pandemic which are clear, reasonable, and relevant throughout the Department’s many contexts and which can be used by faculty to guide teaching, learning and feedback
Phase 2: ‘Input & Clarity check’ *n* = 12	Competencies added, merged, line edited and deleted as directed by reviewers Comment arbitration by two educational leaders from phase 1	*Decisions made by the PG leaders clarified*: *how competencies would be used*: As optional ‘building blocks’ for teaching, learning and assessment. They represent language agreed upon by faculty and residents related to the current crisis, useful for planning educational curriculum, focussing teaching and learning, supporting learners and document attainment of specific competence during these unusual times *how competencies would not be used*: as barriers to residents graduating *how they would be assessed*: No new assessment tools would be created – existing formative and summative tools would be used (Field Notes, end of rotation evaluations)
Phase 3: ‘Broad Input and Dissemination’ *n* = 24	A final list of 33 competencies created across all CanMEDS-FM roles including 8 related to the Professional roles, 7 related to the Health Advocate role, 6 to the Family Medicine Expert role, 3 related to the Communicator, Scholar, Collaborator, and Leader roles	Relevance to those impacted by this beyond resident and faculty leaders was sought PG directors across Canada articulated similar needs simultaneously Sharing and co-development of this educational resource was planned to reduce duplication of efforts in multiple centres

## Discussion

During a pandemic, clinical care is paramount, and consistent with principles of competency-based medical education.^8^^-^^12^ We aimed to analyze and capture the evolving professional, societal, patient and educational needs facing our widely distributed Department. The sweeping reality of the pandemic forced rapid educational change, urgent reflection on professional priorities, roles and identity, and creative adaptation of educational experiences to ensure educational relevance. The current project complements concurrent work by the College of Family Physicians of Canada, guiding virtual supervision of learners.^13^ Rather than a ‘laundry list’ of new educational requirements, the competencies defined here are a resource to use in a wide range of educational contexts to guide teaching, learning and feedback. Although 44 residents and faculty contributed feedback and review of the competencies, the true response rate is undetermined, due to nature of the open invitation to provide input. Another limitation is that these competencies, while vetted by faculty from multiple clinical contexts are largely from a single Canadian university which may limit their immediate applicability in other contexts. The process decision to use existing feedback forms also limits the ease of tracking competency attainment.

## Conclusion

Our process aimed to rapidly engage a broad range of stakeholders to provide a focused educational response to a public health and medical education crisis. Next steps include a program evaluation, after the pandemic. A program evaluation approach will define and judge the success, shortcomings of the pandemic-related changes made across our complex program.^14^ We will be able to judge the impact (intended and unintended) and merit of this rapid medical education pivot with clear questions (eg “Were the competencies attained?” “Were they adequate?” “Are there unmet faculty and residents needs?”) answered through a review of existing data sources (eg formative and summative feedback in field notes and end-of-rotation evaluations), and new data sources (interviews, surveys).

Bringing together a wide range of educational stake-holders (departmental leaders, teachers, residents) from across the continuum of medical education (from undergraduate to postgraduate education and faculty development portfolios) produced an integrated approach to curriculum design, implementation and evaluation. This unprecedented collaboration across portfolios (and universities) was an unexpected outcome of this project, and serves as a model for engagement and cooperation during less turbulent times.
